# Lithography-free directional control of thermal emission

**DOI:** 10.1515/nanoph-2023-0595

**Published:** 2024-01-10

**Authors:** Mitradeep Sarkar, Maxime Giteau, Michael T. Enders, Georgia T. Papadakis

**Affiliations:** ICFO-Institut de Ciencies Fotoniques, The Barcelona Institute of Science and Technology, 08860 Castelldefels, Barcelona, Spain

**Keywords:** emissivity, absorptivity, surface phonon polaritons, dielectric permittivity

## Abstract

Blackbody radiation is incoherent and omnidirectional, whereas various novel applications in renewable energy require a degree of directional control of a thermally emitted beam. So far, such directional control has required nano-structuring the surface of a thermally emitting material, typically by forming diffraction gratings. This, however, necessitates lithography and usually results in polarization-dependent properties. Here, we derive analytical conditions for highly directional thermal emission using a planar 3-layer structure analogous to a Salisbury screen. We present design rules for maximizing the directionality of such structures. Notably, these design rules pertain to both linear polarizations, thus generalizing the principles of a grating for unpolarized light. We show that the key requirement to achieve such performance is ultra-high quality factor resonances in materials supporting phonon polaritonic modes, as those found in low-dimensional materials. We propose a realistic device based on hexagonal Boron Nitride and predict performances comparable to lithography-based nano-structures.

## 1 Introduction

All objects at near-room temperatures emit thermal radiation predominantly at mid-infrared (IR) frequencies. Gaining control over the spatial and spectral characteristics of thermal emission is central to various applications. These include light and energy harvesting [1], for example in thermophotovoltaic systems [2], [3], contactless temperature regulation [4] and radiative cooling [5], [6]. Various other applications also require spatial and spectral engineering of thermal emission, for example in IR sources [7], [8] and thermal camouflage [9]. Often, for optimal efficiency, simultaneously narrow-band and spatially selective thermal emission is required, for example, in molecular sensing [10], [11], [12], [13], thermophotovoltaics [14], and others.

Nonetheless, blackbody thermal emission is spectrally broad and spatially diffuse. Photonic design is typically employed to narrow the spectral characteristics [15], [16] and control the directionality [17] of thermal emission, often done simultaneously [18], [19]. Various nanostructures have been proposed ranging from diffraction gratings [7], [20] to mid-IR antennas [21], [22], multi-layered films and one-dimensional photonic crystals [23], [24], [25], three-dimensional resonators [26], [27], [28], and metasurfaces [29]. The principle of operation of many of these motifs and other elaborate designs [30], [31], [32] relies on the operation principle of a diffraction grating. In particular, one can thermally excite surface phonon polaritons (SPhP) on the surface of a polar dielectric material [33], [34], and, as Greffet et al. experimentally demonstrated in [19], these SPhPs can be diffracted into propagating far-field electromagnetic modes at specific angles, via a grating, thus enabling directional control. The results in [19] can be generalized to any material with a phonon resonance throughout the IR-THz range [35], as well as to plasmonic media at frequencies below the plasma frequency, via the excitation of surface plasmon polaritons [36].

Such phonon or plasmon polaritonic modes, however, occur solely for transverse magnetic (TM) polarization [37]. Therefore, the aforementioned concept of achieving directional control via diffracting surface polaritonic modes to the far-field using a grating or related nano-structure is often constrained to one linear polarization. Additionally, such nano-structures entail lithographic patterning and are often subject to scaling-up challenges.

Recent works have reported directional thermal emission in planar configurations [38], [39], [40], [41]. In [38], a degree of directional control was reported for a planar heterostructure composed of gradient epsilon-near-zero (ENZ) materials, nonetheless this pertained to a broad emission spectrum. In [39], it was theoretically shown that ENZ films on a reflector can steer a thermally emitted beam, but the effect is limited to TM polarization. In [40], a one-dimensional photonic crystal that enables high directionality was reported. To achieve this directionality, a large number of periods of alternating layers is required. In [41], by employing strong optical anisotropies, it was shown that one can use the Brewster’s condition to achieve directionality, however this concept pertains to near-grazing angles of emission. In all the above, the reported directionality remains considerably inferior to that of a grating [19].

**Figure 1:**
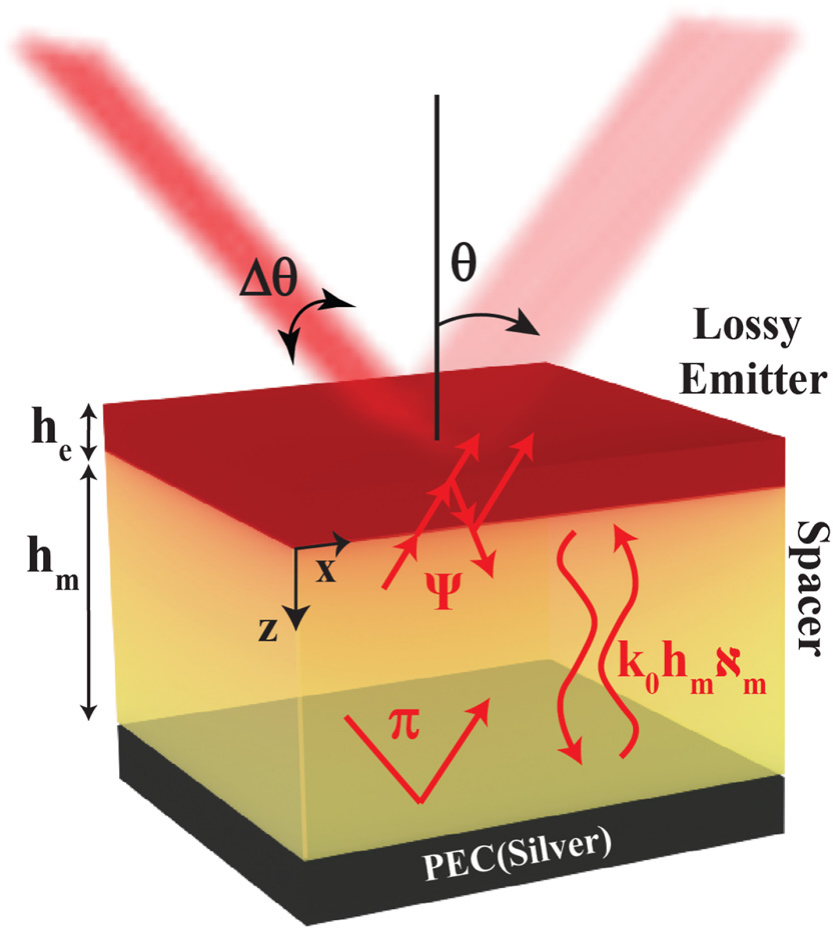
Schematic of the planar structure comprising of a lossy emitter (permittivity *ϵ*_e_) and a lossless spacer (permittivity *ϵ*_*m*_) on a back-reflector, modeled as a perfect electric conductor (PEC). The phase acquired upon reflection onto each interface and due to propagation within a layer are indicated in red. Directional thermal emission at a particular angle *θ* with angular spread Δ*θ* is achieved when the phase matching condition of Eq. (2) is satisfied.

Here, we revisit the concept of a Salisbury screen as a thermal emitter [42]. A Salisbury screen is a three-layer planar hetero-structure constructed of an optically thin lossy material on a thick transparent spacer with a back-reflector, as shown in Figure 1. In this device, the only layer that emits thermal radiation is the top one, as the spacer is lossless and the back-reflector is considered as a perfect electric conductor (PEC). Thus, henceforth, the top layer is termed as the “emitter”. On resonance, the Salisbury screen yields near-unity emissivity due to constructive interference [43]. Although the objective of the original Salisbury screen is not associated with directional control, here we show that, with appropriate engineering of the emitter layer, highly directional thermal emission can be achieved, similar to what has been reported with grating structures [19]. In contrast to gratings, however, the directionality of these Salisbury motifs is preserved for both linear polarizations. The origin of this response is the polar nature of the emitter, which introduces high-permittivity mid-IR resonances [33].

These resonances arise due to phonon polariton modes that can exhibit ultra-long lifetimes, in which cases the material has a very high quality factor (*Q*) [33]. We show that *this* is the mere requirement for achieving strong directionality. These 3-layer hetero-structures can compete and even surpass the performance of nano-structured geometries, provided the emitter has an ultra-high-*Q*, which is often found in low-dimensional materials that support long-lived mid-IR polaritons [35].

## 2 Theoretical formalism

**Figure 2:**
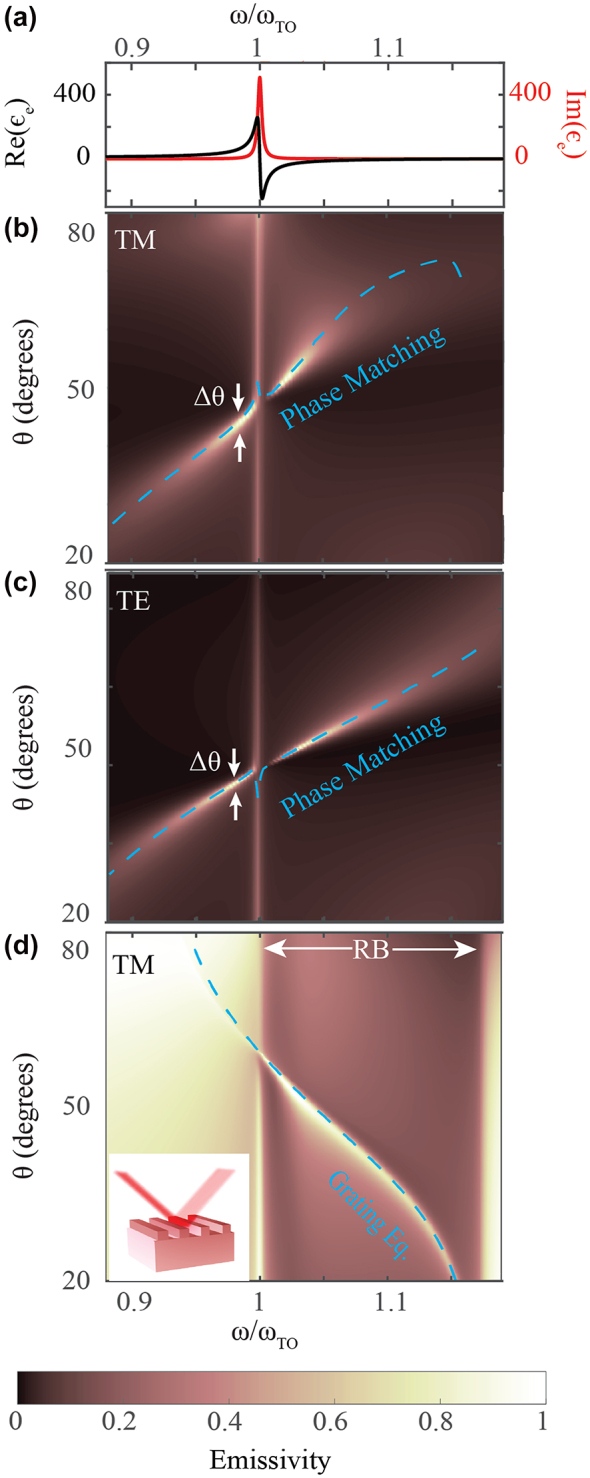
Angular and spectral response of the Salisbury screen and grating structures. (a) The real Re{*ϵ*_e_} (left axis) and imaginary Im{*ϵ*_e_} (right axis) parts of the dielectric permittivity of the lossy emitter (*Q* = 300). (b) The calculated emissivity (*E* = 1 − *R*) as a function of *ω* and *θ* for the planar structure for TM polarized light (*h*_e_/*λ*_TO_ = 0.03, *h*_*m*_/*λ*_TO_ = 0.7). (c) The same as (b) for TE polarized light. The phase matching condition is shown with the blue dashed curves in panels (b) and (c). (d) The same for a grating with grating period *P*/*λ*_TO_ = 0.53 on a semi-infinite polar material with *Q* = 300 for TM polarized light. The grating equation is shown with the blue dashed curve. The Reststrahlen band (RB) in panel d is marked by horizontal white arrows.

We describe the polar material representing the emitting layer in Figure 1 with a dielectric permittivity modeled with a standard Lorentz oscillator [44]:

(1) $$\epsilon_{\text{e}}=\epsilon_{\inf}\left[1+\frac{\omega_{\text{LO}}^{2}-\omega_{\text{TO}}^{2}}{\omega_{\text{TO}}^{2}-i\gamma \omega -\omega^{2}}\right].$$

where *ϵ*_inf_ denotes the high-frequency permittivity and *γ* the damping factor of the phonon polariton mode. The frequencies *ω*_TO_ and *ω*_LO_ correspond to the transverse and longitudinal phonons of the crystal, denoted as TO and LO henceforth. We define the quality factor of the resonance described in Eq. (1) as *Q* = *ω*_TO_/*γ* [33]. For large *Q*, in the frequency range between *ω*_TO_ and *ω*_LO_, Re{*ϵ*_e_} becomes negative. Within this band, often termed the Reststrahlen band (RB), the material displays anomalous dispersion and SPhPs can be excited. The real and imaginary parts of Eq. (1) are shown in Figure 2a, for *Q* = 300, and it can be seen that they resonate near *ω*_TO_.

For maximal performance, we ought to simultaneously maximize the emissivity of the structure at a given zenith angle *θ*, and minimize the angular spread of the emitted beam, Δ*θ*. Therefore, at a given *θ*, we aim to minimize reflectance, for which destructive interference of light escaping the structure must be ensured. Considering that light accumulates a phase of *π* upon reflection at the PEC and a phase of Ψ upon double pass within and reflection from the lossy emitter (Figure 1), a phase-matching condition can be written as [40]:

(2) $$2k_{0}h_{m}{ℵ}_{m}+\pi +\Psi =2l\pi ,$$

where *l* is an integer denoting the interference order, *k*_0_ = 2*π*/*λ*, with *λ* the wavelength of light, *h*_*m*_ is the thickness of the spacer, and ${ℵ}_{m}=\sqrt{\epsilon_{m}-\sin^{2}\theta }$ , with the spacer’s permittivity being *ϵ*_*m*_. In the conventional Salisbury screen notion, the emitter is considered ultra-thin, for which Ψ = 0. Nonetheless, in the present analysis, this condition is relaxed.

By considering that the emitter layer is isotropic, for which *ϵ*_e_ is a scalar, and for incidence from air, one can derive the complex reflection coefficients *r*_TE_ and *r*_TM_, for transverse electric (TE) and transverse magnetic (TM) polarizations, respectively, for this three-layered structure (Figure 1). For TM polarization, the following expression is obtained:

(3) $$r_{\text{TM}}=\frac{\cos\theta \left[1-\frac{\epsilon_{\text{e}}{ℵ}_{m}}{\epsilon_{m}{ℵ}_{\text{e}}}T_{\text{e}}T_{m}\right]+i\left[\frac{{ℵ}_{\text{e}}}{\epsilon_{\text{e}}}T_{\text{e}}+\frac{{ℵ}_{m}}{\epsilon_{m}}T_{m}\right]}{\cos\theta \left[1-\frac{\epsilon_{\text{e}}{ℵ}_{m}}{\epsilon_{m}{ℵ}_{\text{e}}}T_{\text{e}}T_{m}\right]-i\left[\frac{{ℵ}_{\text{e}}}{\epsilon_{\text{e}}}T_{\text{e}}+\frac{{ℵ}_{m}}{\epsilon_{m}}T_{m}\right]},$$

where *T*_e_ = tan(*h*_e_ *k*_0_ *ℵ*_e_), *T*_*m*_ = tan(*h*_*m*_ *k*_0_ *ℵ*_*m*_), ${ℵ}_{\text{e}}=\sqrt{\epsilon_{\text{e}}-\sin^{2}\theta }$ and $n_{\text{e}}=\sqrt{\epsilon_{\text{e}}}$ is the refractive index of the emitter layer. The corresponding expression for TE polarization is shown in the Supplementary Material, Section 1.

To maximize the emissivity, **(A)** the reflection coefficient has to be minimized. By setting *r*_TM_ to 0, through Eqs. (2) and (3), we express the phase acquired in the lossy emitter for unity emissivity:

(4) $$\Psi_{\text{TM}}=Re\left(\pi -2\arctan\left[\frac{\epsilon_{m}{ℵ}_{\text{e}}}{\epsilon_{\text{e}}{ℵ}_{m}}\frac{\epsilon_{\text{e}}\cos\theta +i{ℵ}_{\text{e}}T_{\text{e}}}{\epsilon_{\text{e}}T_{\text{e}}\cos\theta -i{ℵ}_{\text{e}}}\right]\right).$$

To achieve directional control of the emissivity, **(B)** the derivative of the reflection coefficient with respect to *θ* needs to be maximized. We show in Supplementary Material, Section 3, that conditions **(A)** and **(B)** can be satisfied in the following two limiting cases: Re(*n*_e_) ≫ Im(*n*_e_) or for Im(*n*_e_) ≫ Re(*n*_e_). In both cases, the requirement for directional and simultaneously maximal emissivity reduces to $h_{m}=\frac{\lambda }{2{ℵ}_{m}}$ , thus requiring *T*_*m*_ ≈ 0. In both cases: Re(*n*_e_) ≫ Im(*n*_e_) and Im(*n*_e_) ≫ Re(*n*_e_), the condition **(A)** for maximal emissivity is reduced to:

(5) $$1-\frac{{ℵ}_{m}T_{m}n_{\text{e}}T_{\text{e}}}{\epsilon_{m}}+\frac{i}{\cos\theta }\frac{T_{\text{e}}}{n_{\text{e}}}=0,$$

while the condition **(B)** for maximal variation of the emissivity as a function of angle reduces to:

(6) $$\left[\frac{k_{0}h_{m}n_{\text{e}}T_{\text{e}}}{2\epsilon_{m}}\right]\sin2\theta \to \infty .$$

The expressions in Eq. (6) can serve as a design rule for selecting the spacer layer’s permittivity and thickness. First, when the spacer layer is removed (*h*_*m*_ = 0), Eq. (6) cannot be satisfied. Hence, the configuration where the lossy emitter is placed directly on the PEC cannot yield directional control, although unity emissivity can be achieved [45] through Eq. (5). Second, from Eq. (6) it is evident that strong directionality requires a spacer layer with small *ϵ*_*m*_. For this reason, henceforth, we set *ϵ*_*m*_ = 1 throughout the paper. Third, Eq. (6) is satisfied when *n*_e_ *T*_e_ → ∞. In this limit, the phase acquired in the lossy emitter given by Eq. (4) reduces to Ψ_TM_ = *π*. To summarize, directional and maximal thermal emissivity requires a quarter-wave emitter (Ψ = *π*) and a half-wave spacer $\left(h_{m}=\frac{\lambda }{2{ℵ}_{m}}\right)$ .

We note that the conventional Salisbury screen with an optically thin emitter satisfies *h*_e_ ≈ 0 for which Ψ = 0 (Eq. (4)). This yields *h*_*m*_ = *λ*/4*ℵ*_*m*_ from Eq. (2), which is the well-known Fabry–Perot condition for constructive interference in a thick dielectric film for any angle *θ*. The traditional notion of a Salisbury screen pertains to this regime [46], [47], for which there is no phase advancement in the lossy emitter and thus no directional control is possible. In contrast, here, we consider the regime where the emitting layer is *not* optically thin (Ψ = *π*, Eq. (2)) and demonstrate that, in that case, thermal emission can be highly directional.

In particular, using Eq. (3), we show in Figure 2b and c for TM and TE polarization, respectively, the emissivity (*E*_TM∕TE_ = 1 − *R*_TM∕TE_) of the three-layer structure as a function of *ω* and *θ*. In these calculations, the emitter material is considered to have the permittivity shown in Figure 2a. The phase-matching condition is superposed onto these plots (dashed blue curve), and it is clear that near-unity emissivity is achieved when the phase-matching condition is satisfied, for both linear polarizations. As shown, thermal emission is highly directional across the whole spectral range considered.

The only exception occurs at *ω* = *ω*_TO_, for which the real part of the dielectric function (*ϵ*_e_) vanishes. At that frequency, Re(*n*_e_) = Im(*n*_e_), hence the condition for directional emission cannot be satisfied. This can be confirmed by inspecting Figure 2b and c at *ω*_TO_: indeed, at that frequency, the emissivity has no angular dependence. The message of this work is that, by adjusting the thickness of the emitter layer appropriately and operating at *ω* ≠ *ω*_TO_, one can leverage the phase (Ψ) acquired within a polar emitter layer to ensure extremely directional and omnipolarized emission without any lithography.

To appropriately compare our results to the state-of-the-art, in Figure 2d, we calculate, using rigorous coupled wave analysis (RCWA) [48], the emissivity of an optimized grating composed of the same material as the emitting layer (Figure 2a). Indeed, for frequencies within the RB, where SPhP modes are excited, one obtains highly directional and near-unity emissivity. Outside the RB, however, the directional dependence of the emissivity is lost, as expected. The grating equation [19] is superimposed (dashed blue curve) in this plot, and agrees with the points of unity emissivity obtained numerically, as expected. Since the excitation of SPhPs within the emitter is possible only for TM polarization, the grating’s operation is also limited to this polarization. Hence, the emissivity for TE polarization is omitted as it is fully diffuse. With respect to gratings, the 3-layered structure considered here is polarization-independent. This attribute, in addition to the continuous change of central angle of emission as a function of wavelength (Figure 2b and c) and the lack of need for lithography makes this structure relevant for applications in beam steering.

**Figure 3:**
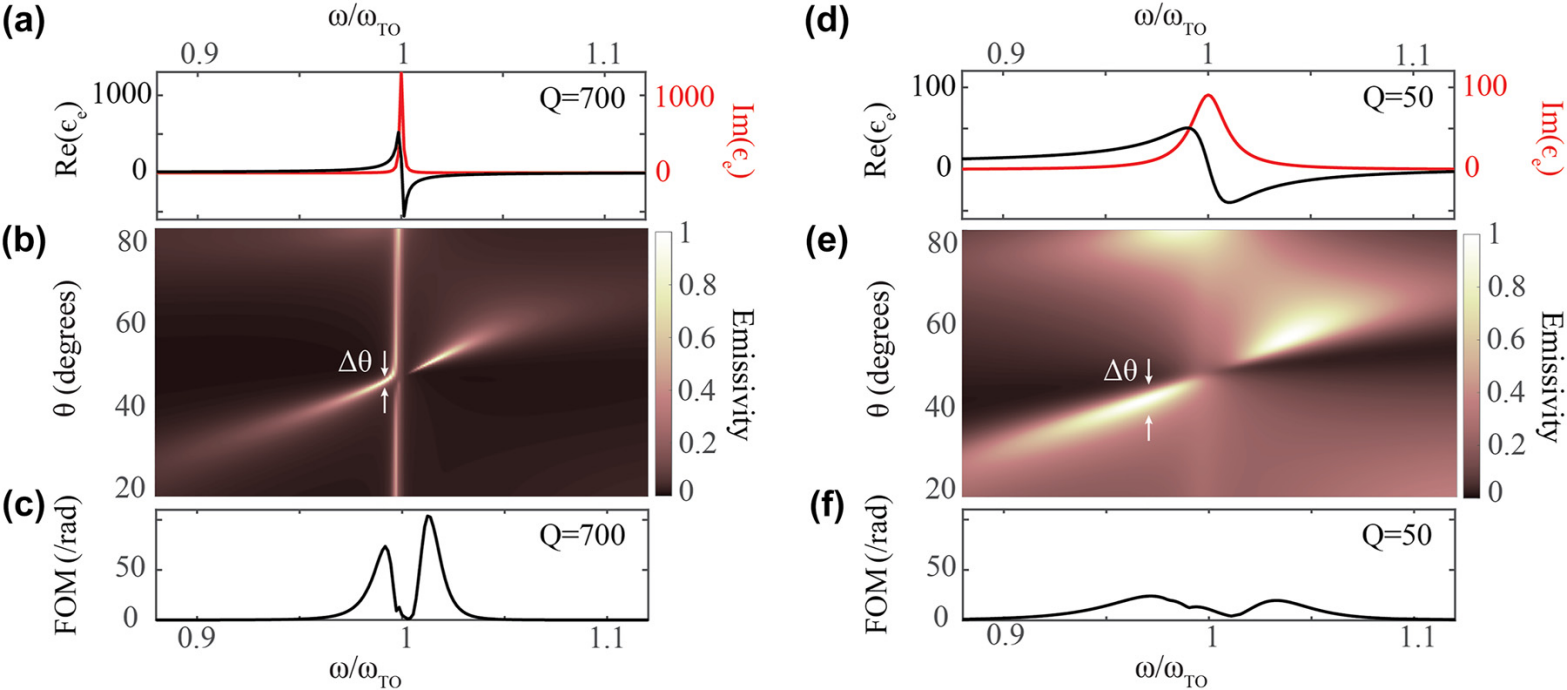
Effect of *Q* on directionality. (a, b) The same as Figure 2a and b for the same structure with *Q* = 700. (c) The calculated FOM for the structure is shown. (d–f) The same as (a–c) with *Q* = 50.

In Figure 3, we present emissivity for two extreme cases: left – for an ultra-high-*Q* of 700 (Figure 3a–c) and right – for a low-*Q* of 50 (Figure 3d–f). The respective permittivities for these two quality factors are shown in Figure 3a–d. As shown, the amplitudes of Re{*ϵ*_e_} and Im{*ϵ*_e_} on resonance are proportional to the material’s quality factor. For all calculations in Figure 3, the same *ω*_TO_, *ω*_LO_, and *ϵ*_inf_ were considered as in 2. It can be seen in Figure 3e that the emissivity is near-unity for a much broader range of angles for *Q* = 50 as compared to *Q* = 700 (Figure 3b). In other words, the angular spread (Δ*θ*), as denoted in Figure 3b and e, is much narrower for *Q* = 700 as compared to *Q* = 50. This demonstrates that, indeed, a larger quality factor yields improved directionality.

To summarize, we comment that, although for thermal emission to occur a certain amount of optical loss is necessary, to achieve strong directionality in these Salisbury-like structures, a low damping rate (*γ* in Eq. (1)) is required. By appropriately adjusting material losses through *γ*, one can achieve strong directionality without any lithography. In the following section, we present design rules and performance metrics for evaluating the performance of these devices.

## 3 Design rules for directional emission

To evaluate and compare the performance of various geometries as directional thermal emitters, we consider two relevant properties: (i) the angular spread of the emissivity, Δ*θ* at each frequency, shown in Figures 2b, 3b and e, and (ii) the contrast between maximum and minimum emissivity at the frequency of operation, defined as $C=\left|R^{\min}\left(\theta \right)-R^{\max}\left(\theta \right)\right|$ . The ratio *C*/Δ*θ* expresses the degree of directionality of a thermally emitted beam. By *C*/Δ*θ* ≈ |*dR*/*dθ*|, we define a figure of merit (FOM) of the directionality as:

(7) $$FOM=\left|\frac{dR}{d\theta }\right|$$

By the definition of Eq. (7), a large FOM suggests highly directional emission with high peak emissivity, such as that achieved by a grating [19]. By contrast, a low FOM indicates either low emissivity or diffuse thermal emission. The FOM at each frequency is evaluated as the maximum value of the derivative of reflectance with respect to *θ*. Thus, the FOM scales as Eq. (6), and, as such, it varies sinusoidally with 2*θ*.

Indeed, *Q* = 700 results in a much higher FOM than *Q* = 50, as can be clearly seen by comparing Figure 3c, and f. As shown in these plots, the FOM has two peaks near *ω* = *ω*_TO_, each peak corresponding to the condition |Re{*ϵ*_e_}| ≫ Im{*ϵ*_e_} on either side of *ω*_TO_. Additionally, as expected, the FOM vanishes at *ω* = *ω*_TO_, since, at that frequency, Im{*ϵ*_e_} ≫ |Re{*ϵ*_e_}| and the interference effects required for directionality are masked by the strong absorption of the emitter material.

**Figure 4:**
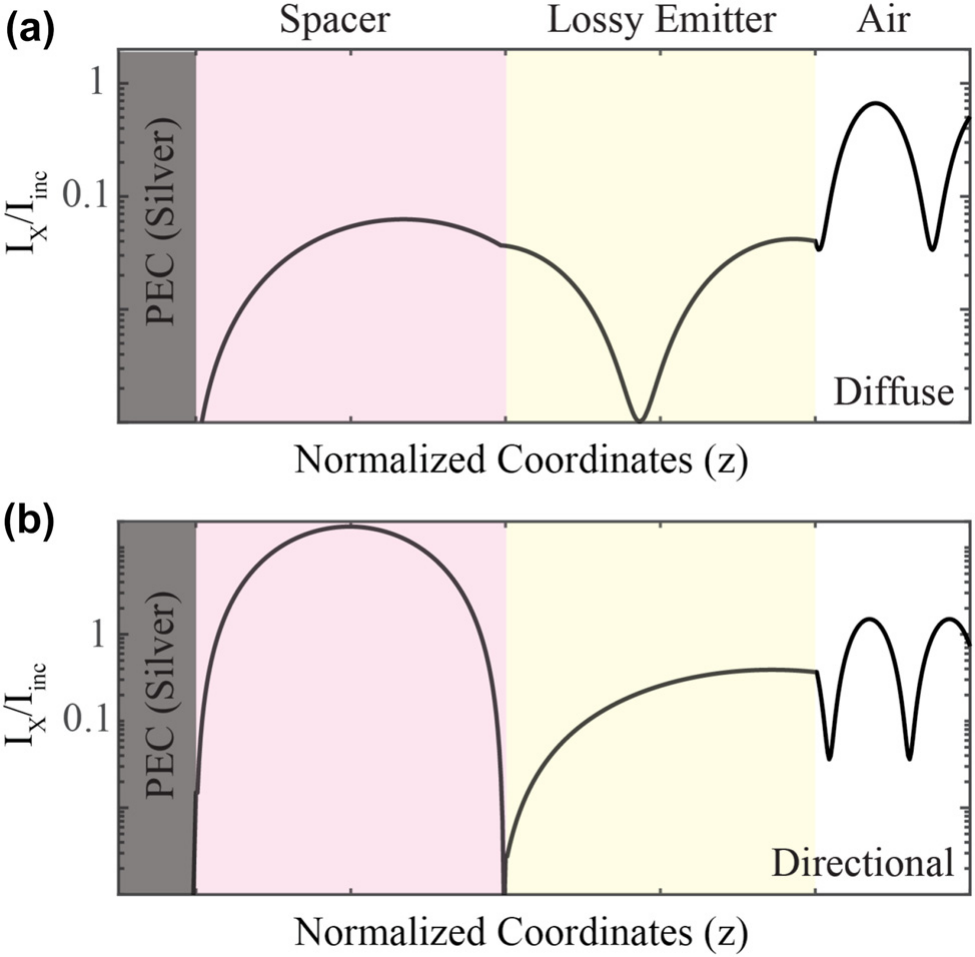
In-plane intensity of electric field profiles for the 3-layer structure shown in Figure 3b, for emitter *Q* = 700 (a) at *ω* ≈ *ω*_TO_ and *θ* = 50°, when thermal emission is diffuse and (b) at *ω* = 0.99 *ω*_TO_ and *θ* = 45°, where the FOM is maximum (marked by white arrows in Figure 3b). The coordinates of each layer’s boundaries (emitter, spacer) are normalized by their thicknesses (*h*_e_, *h*_*m*_), respectively. The field intensity is shown in log scale.

In Figure 4 we present the intensity profiles of the electromagnetic field inside the 3-layer structures for two pairs of (*ω*, *θ*): for diffuse emission at *ω* ≃ *ω*_TO_, and for directional emission, corresponding to *ω* = 0.99 *ω*_TO_ and *θ* = 45° as marked by white arrows in Figure 3b, for the emitter with *Q* = 700. As shown in Figure 4a, in the diffuse case, the lossy emitter contains half a wavelength of the field. Hence the total phase acquired for two passes of the light in the emitter is Ψ ≈ 2*π*. This can be generalized to Ψ ≈ 2*lπ* where *l* = 0, 1, 2… at the edge of the RB (Eq. (2)). This is expected, since at *ω*_TO_, the material acts as an ENZ medium and is highly absorptive.

By contrast, in Figure 4b, it is seen that the field within the emitter, upon a double pass, acquires a phase of Ψ = *π*. In order to satisfy Eq. (2), light in the lossless spacer must acquire a phase of 2*lπ* for the double-pass, which indeed is the case as Figure 4b, where the field experiences two zeros, one at the PEC-spacer interface, and another at the spacer-lossy emitter interface. The condition Ψ = *π* for directional emission is analytically demonstrated in the Supplementary Material, Section 3.

To summarize this section, we have demonstrated that, by appropriately designing the emitter to control the phase acquired within the emitter (Ψ in Eq. (4)), one can achieve either fully diffuse or directional thermal emission. In the next section, we outline the rules for optimizing directional emission.

## 4 Optimization of the planar structure

In the previous sections, we derived analytical conditions for achieving directional thermal emission in the Salisbury screen configuration. We showed that, for obtaining a strong degree of directionality, the emitter layer ought to have a large quality factor, *Q*, for which |Re(*ϵ*_e_)| ≫ Im(*ϵ*_e_). This condition is satisfied on either side of *ω*_TO_. Next, we demonstrate how one can design a directional thermal emitter to emit optimally at a particular zenith angle *θ*, given an emitter material of permittivity *ϵ*_e_(*ω*). As discussed before (Eq. (6)), the spacer layer is chosen to be air.

Upon selection of the spacer layer’s permittivity, given a certain angle of emission *θ* and the material of the emitter layer *ϵ*_e_, the emitter height should be selected. To warrant directionality and critical coupling for maximizing emissivity [49], from Eq. (3), one can derive the optimal height of the emitter within the RB as well as for *ω* < *ω*_TO_. As shown in Supplementary Material, Section 3, for these two frequency ranges, one obtains

(8) $$\begin{array}{llll} h_{\text{e}} & =\frac{\lambda }{8\pi Im\left\{\sqrt{\epsilon_{\text{e}}}\right\}}arcsinh\frac{2}{\cos\theta Re\left\{\sqrt{\epsilon_{\text{e}}}\right\}} & & :\omega >\omega_{\text{TO}}\\ h_{\text{e}} & =\frac{\lambda }{4Re\left\{\sqrt{\epsilon_{\text{e}}}\right\}} & & :\omega <\omega_{\text{TO}} \end{array}$$

For the emitter thickness given by Eq. (8), the emissivity is always maximized however the FOM is maximum only for the frequencies where Ψ = *π* from Eq. (4). This is discussed in detail in Supplementary Material, Section 3. Consequently, the spacer height, *h*_*m*_, can be computed using Eqs. (2) and (4) with the corresponding value of *h*_e_.

**Figure 5:**
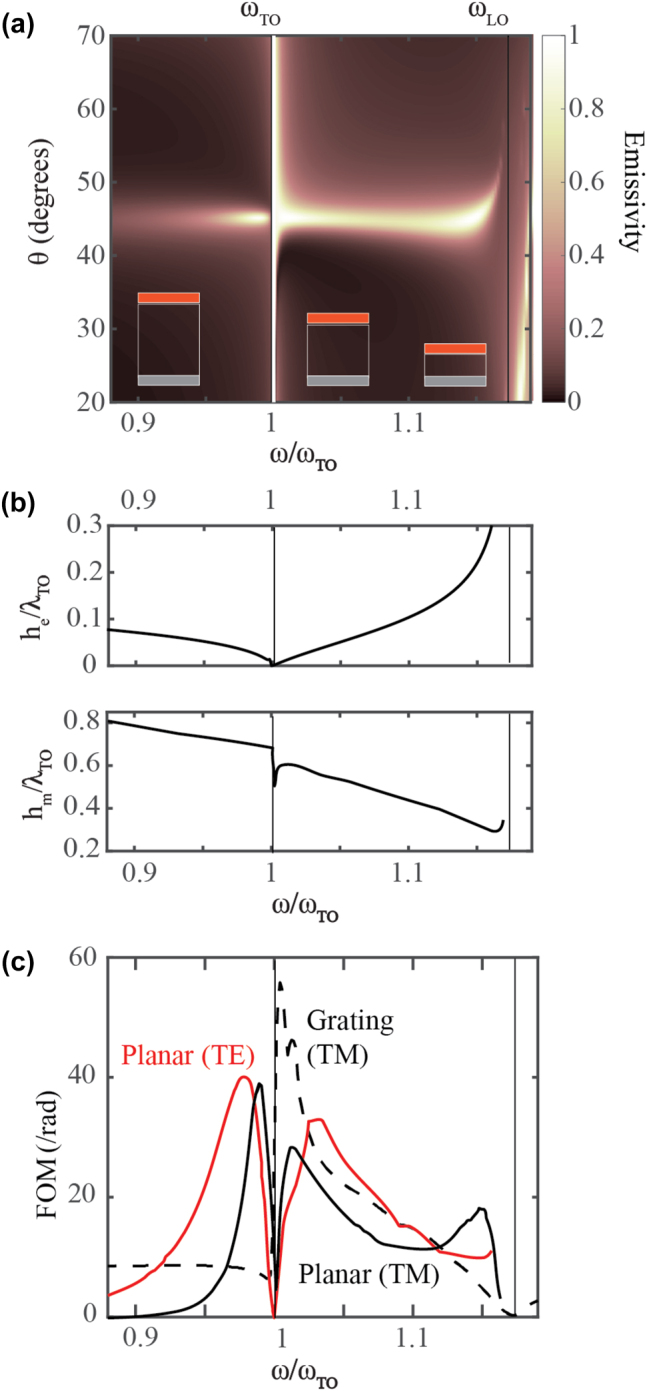
Optimization of the Salisbury screen configuration at each frequency. (a) The calculated emissivity (*E* = 1 − *R*) as a function of *ω* (normalized to *ω*_TO_) and *θ* for the planar structures optimized at each *ω* for TM polarized light. Schematics of the three-layer structures are shown as insets. (b) The emitter and spacer heights (*h*_e_ and *h*_*m*_) normalized to the wavelength of the TO phonon resonance (*λ*_TO_), optimized at each frequency to have directional emission at *θ* = 45°. (c) The FOM calculated by Eq. (7) for the optimized grating (shown in Supplementary Material, Section 4) for TM polarization (black dashed) and for the planar structure (shown in (a)) for TM and TE polarizations (black solid and red, respectively). The RB (between *ω*_TO_ and *ω*_LO_) is marked by vertical black lines. *Q* = 300 for the emitter in this figure.

As a demonstration of the aforementioned procedure, for the considered emitter of *Q* = 300 and for *ϵ*_*m*_ = 1 for all frequencies, we optimize the planar structure for TM polarization. We choose the central emission angle to be *θ* = 45°. In Figure 5a, we show the emissivity map as a function of (*ω*, *θ*), where the optimization is carried out separately for each frequency. The corresponding height of the emitter layer, *h*_e_, and spacer layer, *h*_*m*_, are shown in the top and bottom panels of Figure 5b, respectively. In Figure 5c, we present the corresponding FOM for both linear polarizations which is very high due to the optimization. Importantly, we also present the calculated FOM for TM polarization for an optimized diffraction grating composed of the same material as the emitter layer of the 3-layer structure, shown with the dashed black curve. As expected, the FOM of the grating only increases within the RB of the emitter. It is evident that the directionality of the planar structure is comparable to that of the grating.

The Salisbury screen does not assure broadband operation. The purpose of Figure 5a is to emphasize that the structure can be optimized for directional emission at each frequency both inside and outside the RB with the only exception of *ω* = *ω*_TO_. The design rules are independent of material dispersion and can be performed for all angles *θ*. For each optimized structure, the frequency response should be inferred from Figures 2 and 3.

As a final step, we seek realistic materials for a 3-layer Salisbury screen that can serve as a directional thermal emitter within the mid-IR range. For the emitter layer, we consider hexagonal boron nitride (hBN) with permittivity obtained from Caldwell et al. [50]. hBN is selected because it has a very sharp dielectric permittivity resonance at the wavelength of 7.3 μm due to an in-plane phonon polariton, with *Q* = 274. The out-of-plane phonon polariton is irrelevant here, but, as a reference, we note that it has a permittivity of 1.65 in the spectral range of interest. The spacer layer is considered as air, while silver is considered as the back-reflector, with its permittivity taken from Rakić et al. [51]. Without loss of generality, we optimize the structure to have a peak emission *θ* = 45° for TM polarized light.

**Figure 6:**
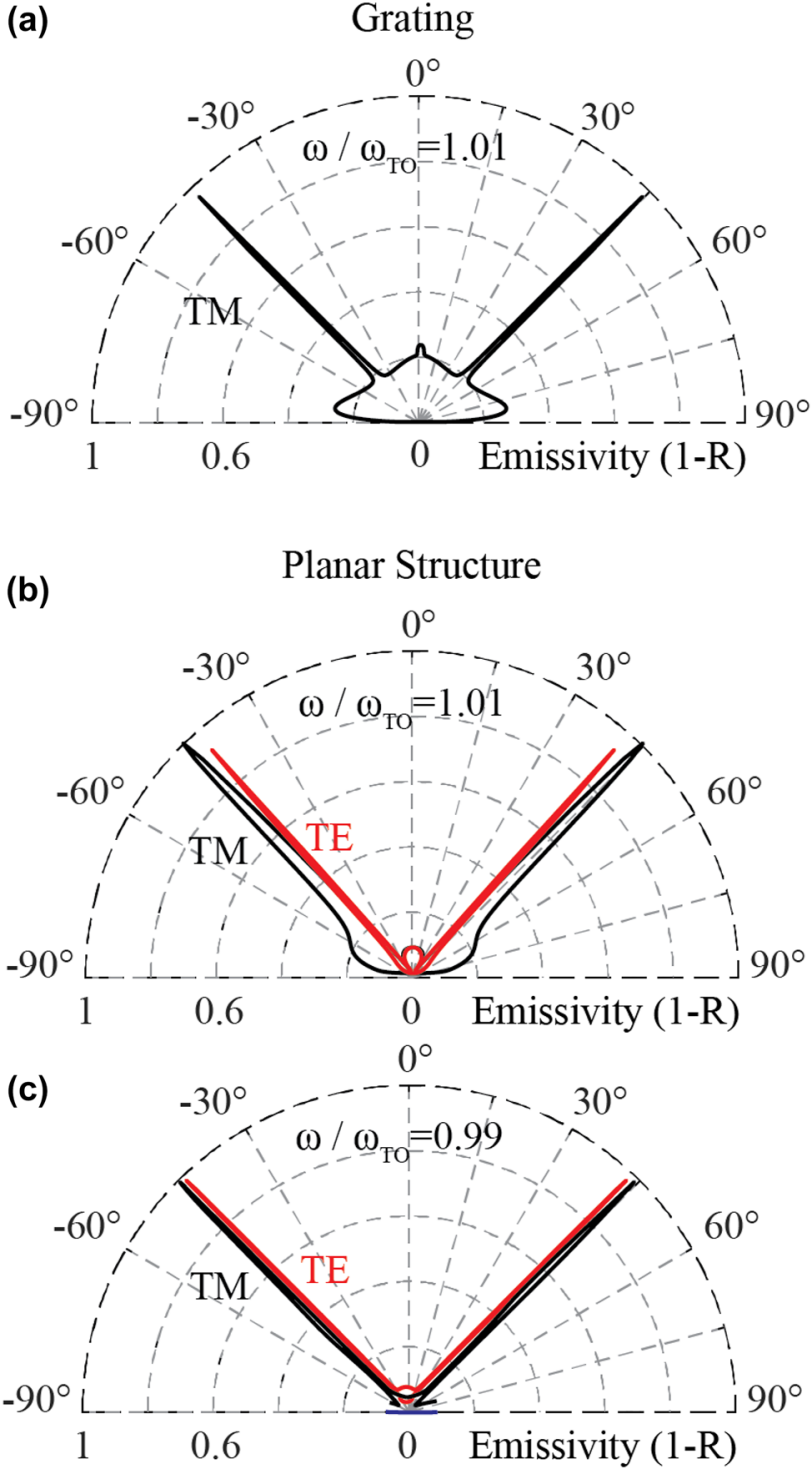
Angular response of the optimized structures. (a) The polar plot for TM polarized emission from the hBN grating structure for a frequency inside the RB (*ω*/*ω*_TO_ = 1.01). (b) Polar plot of the emissivity of an optimized planar structure for both TM and TE polarized light, (black and red, respectively) at the same frequency as in panel (a). (c) The same as (b) for a frequency outside the RB (*ω*/*ω*_TO_ = 0.99). The back-reflector of the planar structure is silver.

The polar plots of the emissivity are shown in panels b and c of Figure 6 for frequencies 1.01*ω*_TO_ and 0.99*ω*_TO_, respectively, for both linear polarizations. As can be seen, ultra-narrow lobes with high contrast, resembling those of a directional antenna, are achieved, without any need for lithography. For reference, in Figure 6a, we present the polar map of a diffraction grating, also composed and optimized for hBN. The lobes shown in Figure 6a only apply to TM polarization, as explained earlier. Figure 6 indeed demonstrates that, with careful design of a Salisbury screen and by selecting emitting materials with ultra-high *Q*, one can achieve strongly directional and omnipolarized thermal emission or absorption. Apart from hBN, other candidates for the emitter layer are SiC, *α*-MoO_3_, and III–V materials with high Q like GaAs, InP and AlAs that have high *Q* factors [33].

## 5 Conclusions

In this article, we demonstrate an approach for achieving strong spatial and spectral control of thermal emission using the traditional Salisbury screen. Unlike most motifs, where directional control is achieved independently for the two linear polarizations, we demonstrate that these 3-layered heterostructures can support highly directional emission for both polarizations simultaneously. We present a simple geometry that interprets the principle of operation of these motifs using the phase-matching condition, and showcase that materials with long-lived phonon polariton resonances are suitable for directional emission.

We show that, although optical losses are a pre-requisite for thermal emission, for higher directionality of the emitted light, the condition |Re{*ϵ*_e_}| > Im{*ϵ*_e_} must be satisfied. This is possible on either side of *ω*_TO_ but not at *ω* = *ω*_TO_, where the emission is always diffuse. The directional emission can be passively tuned over any spectral range where the phonon resonances occur. The structure presented here does not require expensive lithography or synthesis techniques, but mere thin-film deposition.

## Supplementary Material

Supplementary Material Details
