# Supplementary material for: Lithography-free directional control of thermal emission
Source: Nanophotonics. 2024 Jan 10;13(5):763–71. doi: 10.1515/nanoph-2023-0595 (PMC11502043; doi:10.1515/nanoph-2023-0595)
Supplement: Supplementary file 1 — Supplementary Material Details [file j_nanoph-2023-0595_suppl_001.pdf]

Mitradeep Sarkar, Maxime Giteau, Michael Enders, and Georgia Papadakis*

Lithography-free directional control of thermal emission : Supplementary Material

1 Derivation of the analytical expressions for reflectivity

The system consists of an incident medium with permittivity $\epsilon_1 = n_1^2 = 1$, a substrate with $\epsilon_2 = n_2^2$ and two thin layers of thickness h_e and h_m with permittivity ϵ_e and ϵ_m. Both media are considered to be anisotropic with diagonal terms ϵ_x, $\epsilon_y = \epsilon_x/\alpha_Y^2$ and $\epsilon_z = \epsilon_x/\alpha_Z^2$. The reflection ($r_{p/s}$) and transmission ($t_{p/s}$) of the system can be written in terms of the transfer matrices as

$$\begin{bmatrix} t_s \\ t_p \\ 0 \\ 0 \end{bmatrix} = K_2^{-1} M_m M_e K_1 \begin{bmatrix} a_s \\ a_p \\ r_s \\ r_p \end{bmatrix} \tag{S1}$$

where a_s and a_p are the TE and TM polarized incident fields. K_2 and K_1 are the boundary conditions for the interfaces between the medium m and substrate and medium e and incident medium respectively.

$$K_{2/1} = \begin{bmatrix} 0 & -\cos\theta & 0 & cos\theta \\ 1 & 0 & 1 & 0 \\ -\frac{\sqrt{\epsilon_{2/1}}}{\eta_0}\cos\theta & 0 & \frac{\sqrt{\epsilon_{2/1}}}{\eta_0}\cos\theta & 0 \\ 0 & -\frac{\sqrt{\epsilon_{2/1}}}{\eta_0} & 0 & -\frac{\sqrt{\epsilon_{2/1}}}{\eta_0} \end{bmatrix} \tag{S2}$$

with $\eta_0 = \sqrt{\mu_0/\epsilon_0}$. The transfer matrices along with the boundary conditions at the interfaces for the two thin media with thickness h can be written as $M = e^{iPh}$, with

$$P = \omega \begin{bmatrix} 0 & 0 & 0 & \eta_0^2 - \eta_0^2 \sin^2\theta/\epsilon_z \\ 0 & 0 & -\eta_0^2 & 0 \\ 0 & -\epsilon_y + \sin^2\theta & 0 & 0 \\ \epsilon_x & 0 & 0 & 0 \end{bmatrix} \tag{S3}$$

After matrix exponential, M can be written as

$$\begin{bmatrix} \cos(k_0 h \aleph_Z) & 0 & 0 & i\frac{\eta_0 \aleph_Z \sin(k_0 h \aleph_Z)}{\epsilon_x} \\ 0 & \cos(k_0 h \aleph_Y) & -i\frac{\eta_0 \sin(k_0 h \aleph_Y)}{\aleph_Y} & 0 \\ 0 & -i\frac{\aleph_Y \sin(k_0 h \aleph_Y)}{\eta_0} & \cos(k_0 h \aleph_Y) & 0 \\ i\frac{\epsilon_x \sin(k_0 h \aleph_Z)}{\eta_0 \aleph_Z} & 0 & 0 & \cos(k_0 h \aleph_Z) \end{bmatrix} \tag{S4}$$

Performing the matrix multiplication and rearranging the terms one can derive the following explicit relations for the reflectivity

$$r_P = \frac{[F_{1P} C_e C_m + F_{2P} S_e S_m] + i\,[F_{3P} S_e C_m + F_{4P} S_m C_e]}{[G_{1P} C_e C_m - G_{2P} S_e S_m] - i\,[G_{3P} S_e C_m + G_{4P} S_m C_e]}$$
$$r_S = \frac{[F_{1S} C_e C_m + F_{2S} S_e S_m] + i\,[F_{3S} S_e C_m + F_{4S} S_m C_e]}{[G_{1S} C_e C_m - G_{2S} S_e S_m] - i\,[G_{3S} S_e C_m + G_{4S} S_m C_e]} \tag{S5}$$

Mitradeep Sarkar, Maxime Giteau, Michael Enders, Georgia Papadakis, ICFO-Institut de Ciencies Fotoniques, The Barcelona Institute of Science and Technology, 08860 Castelldefels (Barcelona), Spain e-mail: georgia.papadakis@icfo.eu

where

$$F_{1P} = 1 - \frac{n_1 \cos\theta_T}{n_2 \cos\theta}, F_{1S} = \frac{n_1 \cos\theta}{n_2 \cos\theta_T} - 1,$$
$$G_{1P} = 1 + \frac{n_1 \cos\theta_T}{n_2 \cos\theta}, G_{1S} = \frac{n_1 \cos\theta}{n_2 \cos\theta_T} + 1,$$

$$F_{2P} = \frac{n_1 \cos\theta_T n_{xm}^2 \aleph_{Ze}}{n_2 \cos\theta n_{xe}^2 \aleph_{Zm}} - \frac{n_{xe}^2 \aleph_{Zm}}{n_{xm}^2 \aleph_{Ze}}, F_{2S} = \frac{\aleph_{Ye}}{\aleph_{Ym}} - \frac{n_1 \cos\theta \aleph_{Ym}}{n_2 \cos\theta_T \aleph_{Ye}},$$
$$G_{2P} = \frac{n_1 \cos\theta_T n_{xm}^2 \aleph_{Ze}}{n_2 cos\theta n_{xe}^2 \aleph_{Zm}} + \frac{n_{xe}^2 \aleph_{Zm}}{n_{xm}^2 \aleph_{Ze}}, G_{2S} = \frac{\aleph_{Ye}}{\aleph_{Ym}} + \frac{n_1 cos\theta \aleph_{Ym}}{n_2 \cos\theta_T \aleph_{Ye}},$$

$$F_{3P} = \frac{n_1 \aleph_{Ze}}{n_{xe}^2 \cos\theta} - \frac{n_{xe}^2 \cos\theta_T}{n_2 \aleph_{Ze}}, F_{3S} = \frac{\aleph_{Ye}}{n_2 \cos\theta_T} - \frac{n_1 \cos\theta}{\aleph_{Ye}}$$
$$G_{3P} = \frac{n_1 \aleph_{Ze}}{n_{xe}^2 \cos\theta} + \frac{n_{xe}^2 \cos\theta_T}{n_2 \aleph_{Ze}}, G_{3S} = \frac{\aleph_{Ye}}{n_2 \cos\theta_T} + \frac{n_1 \cos\theta}{\aleph_{Ye}}$$

$$F_{4P} = \frac{n_1 \aleph_{Zm}}{n_{xm}^2 \cos\theta} - \frac{n_{xm}^2 \cos\theta_T}{\aleph_{Zm}}, F_{4S} = \frac{\aleph_{Ym}}{n_2 \cos\theta_T} - \frac{n_1 \cos\theta}{\aleph_{Ym}}$$
$$G_{4P} = \frac{n_1 \aleph_{Zm}}{n_{xm}^2 \cos\theta} + \frac{n_{xm}^2 \cos\theta_T}{n_2 \aleph_{Zm}}, G_{4S} = \frac{\aleph_{Ym}}{n_2 \cos\theta_T} + \frac{n_1 \cos\theta}{\aleph_{Ym}}$$

$$TM: \ C_{e/m} = \cos\left(h_{e/m} k_0 \aleph_{Ze/m}\right), S_{e/m} = \sin\left(h_{e/m} k_0 \aleph_{Ze/m}\right),$$
$$TE: \ C_{e/m} = \cos\left(h_{e/m} k_0 \aleph_{Ye/m}\right), S_{e/m} = \sin\left(h_{e/m} k_0 \aleph_{Ye/m}\right),$$

$n_{z1} \sin(\theta) = n_{z2} \sin(\theta_T)$

Considering $n_1 = 1$ and PEC as the substrate, hence $1/n_2 \approx 0$ we can get the expression for the reflection coefficient (r) as

$$r_{TM} = \frac{\cos\theta \left[1 - \frac{\epsilon_e \aleph_m}{\epsilon_m \aleph_{Ze}} T_e T_m\right] + i \left[\frac{\aleph_{Ze}}{\epsilon_e} T_e + \frac{\aleph_m}{\epsilon_m} T_m\right]}{\cos\theta \left[1 - \frac{\epsilon_e \aleph_m}{\epsilon_m \aleph_{Ze}} T_e T_m\right] - i \left[\frac{\aleph_{Ze}}{\epsilon_e} T_e + \frac{\aleph_m}{\epsilon_m} T_m\right]}$$
$$r_{TE} = \frac{\left[1 - \frac{\aleph_{Ye}}{\aleph_m} T_e T_m\right] + i \cos\theta \left[\frac{1}{\aleph_{Ye}} T_e + \frac{1}{\aleph_m} T_m\right]}{\left[1 - \frac{\aleph_{Ye}}{\aleph_m} T_e T_m\right] - i \cos\theta \left[\frac{1}{\aleph_{Ye}} T_e + \frac{1}{\aleph_m} T_m\right]} \tag{S6}$$

where $T_e = \tan(h_e k_0 \aleph_{Z/Ye})$ for P/S polarizations and $T_m = \tan(h_m k_0 \aleph_m)$ for an isotropic spacer. The terms $\aleph_{Ze} = \sqrt{\epsilon_e - \alpha_Z^2 \sin^2\theta}$ and $\aleph_{Ye} = \sqrt{\epsilon_e - \alpha_Y^2 \sin^2\theta}/\alpha_Y$.

2 Figure of Merit calculations

The FOM is defined as

$$FOM = \frac{Contrast(C)}{AngularSpread(\Delta\theta)} \propto \left|\frac{dR_{TM/TE}}{d\theta}\right| \tag{S7}$$

The reflectivity was calculated using Eq.S5. Contrast was calculated directly from the reflectivity curves as the difference between the maximum and minimum reflectivity values. For the angular spread ($|dR_{TM/TE}/d\theta|$), the derivative of the reflectivity was taken with respect to θ and the maximum value of the derivative was used to calculate the FOM.

This is shown in Fig. S1 for the directional and diffuse cases ($\epsilon_e = 100 + 10i$ and $\epsilon_e = 100 + 343i$ respectively). For both values of ϵ_e, h_m and h_e were optimized to have maximum emissivity (E) at $\theta = 45°$.

The two components of FOM namely the contrast (emissivity) and $|dR_{TM/TE}/d\theta|$ as a function of Re(ϵ_e) and Im(ϵ_e) for TM polarized light is shown in Fig. S1a, S1c and for TE polarization in Fig. S1b, S1d. We see that the contrast does not vary significantly with ϵ_e. Hence the FOM is mainly dominated by the term $|dR/d\theta|$. Also, the condition $|Re(\epsilon_e)| \gg Im(\epsilon_e)$ for higher directionality as discussed in the main paper is evident here.

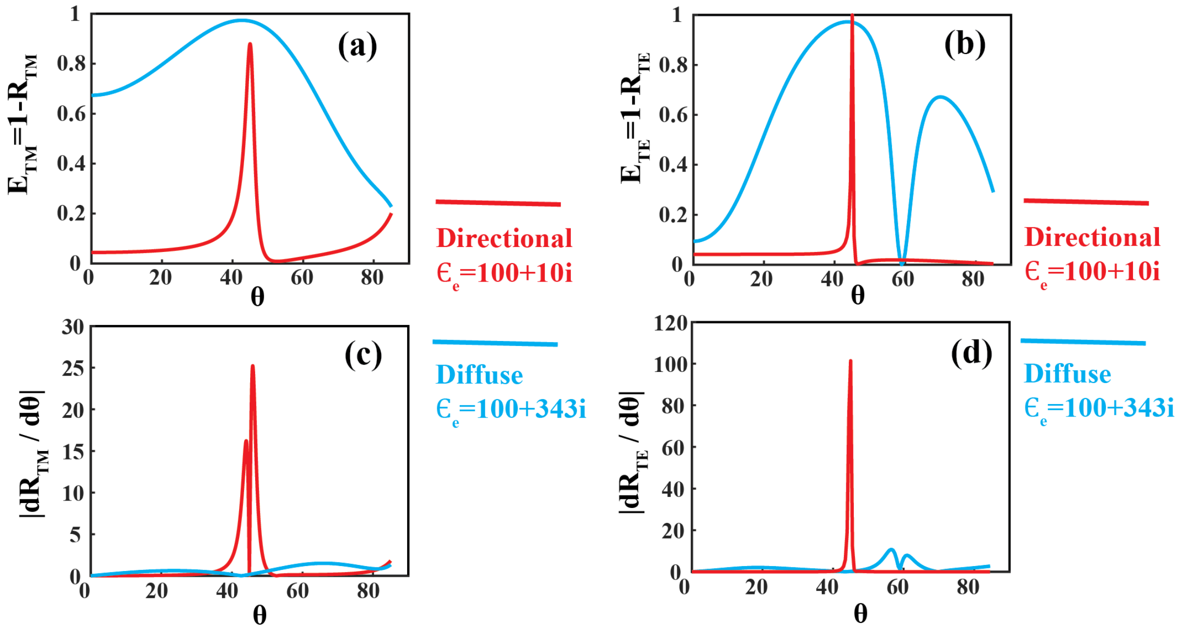

Fig. S1: (a,b) The emissivity curves ($1 - R_{TM}$ and $1 - R_{TE}$ respectively) as a function of θ for directional ($Re(\epsilon_e) > Im(\epsilon_e)$, red) and diffuse ($Re(\epsilon_e) < Im(\epsilon_e)$, blue) emissions. (c,d) The derivatives of (a,b) with respect to θ.

3 Optimization for unity and directional emissivity

The phase matching condition to have constructive interference in the structure is given as

$$2k_0 h_m \aleph_m + \pi + \Psi = 2l\pi \tag{S8}$$

So, the acquired phase on reflection from the lossy material (Ψ) can hence be derived from Eq.S6 and Eq.S8 as

$$\Psi_{TM} = \pi - 2\arctan\left[\frac{\aleph_{Ze}\epsilon_m}{\epsilon_e\aleph_m}\frac{\epsilon_e cos\theta + i\aleph_{Ze}T_e}{\epsilon_e cos\theta T_e - i\aleph_{Ze}}\right] \tag{S9}$$

$$\Psi_{TE} = \pi - 2\arctan\left[\frac{\aleph_m}{\aleph_{Ye}}\frac{\aleph_{Ye} + icos\theta T_e}{\aleph_{Ye}T_e + icos\theta}\right]$$

for TM and TE polarizations respectively. We define the phase acquired in the emitter as the real part of Eq. S16. The phase Ψ was calculated for the structures shown in Fig 3 of the main article for 2 different values of Q and is shown in Fig. S2. We see that outside the RB the peaks of FOM correspond to $\Psi = \pi$ as discussed in the main article. The derivation is as follows.

For high Q materials, $\epsilon_e \gg \sin^2\theta$, thus $\aleph_{Ze/Ye} \approx \sqrt{\epsilon_e} = n_e$. Hence we can write $T_e \approx tan(h_e k_0 n_e)$, where both n_e and T_e are independent of θ.

Writing Eq.S6 as $r = A(\omega,\theta)/B(\omega,\theta)$ we see for directional unitary emission, the numerator

$$A(\omega,\theta) = 1 - \frac{T_m\aleph_m}{\epsilon_m}n_eT_e + i\chi\left(\frac{T_e}{n_e} + \frac{\aleph_m T_m}{\epsilon_m}\right) \tag{S10}$$

must be zero, where $\chi = \cos\theta$ for TE polarization and $\chi = 1/\cos\theta$ for TM polarization. Approximating $\aleph_m \approx n_e$, we can derive the condition presented in Eq. 5 of the main article. For directional emission, an additional condition is that the derivative of r with respect to θ must be maximized. We note that when $A(\omega,\theta) = 0$,

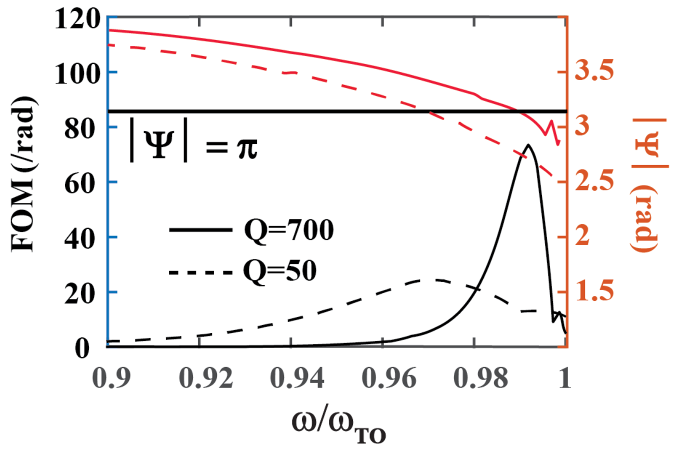

Fig. S2: The FOM for the planar structure for TM polarized light ($h_e/\lambda_{TO} = 0.03$, $h_m/\lambda_{TO} = 0.7$) for 2 different values of Q (black lines, left axis). The corresponding phase acquired in the emitter (Ψ) calculated from Eq.S8 (red lines, right axis). The structures correspond to the color maps of Fig 3 of the main article.

$$\frac{d}{d\theta}\left[\frac{A}{B}\right] = \frac{BA' - AB'}{B^2} = \frac{1}{B}\frac{dA}{d\theta} \tag{S11}$$

From Eq. S10 we get

$$\frac{dA}{d\theta} = \left[\frac{k_0 h_\mathrm{m} n_\mathrm{e} T_\mathrm{e}}{2\epsilon_\mathrm{m}}\right] \sin 2\theta + \frac{i\tan\theta}{\cos\theta}\frac{T_\mathrm{e}}{n_\mathrm{e}} \tag{S12}$$

For directional emissivity, Eq. S12 must be maximized. To achieve this, we consider two alternative approaches that consist in maximizing $n_e T_e$ to increase the angular sensitivity of r.

The term T_e is complex and can be generally written as

$$T_e = tan(\rho + i\beta) = \frac{sin(2\rho) + isinh(2\beta)}{cos(2\rho) + cosh(2\beta)} \tag{S13}$$

where $\rho = k_0 h_e \mathrm{Re}(n_e)$ and $\beta = k_0 h_e \mathrm{Im}(n_e)$. A first strategy consists in minimizing the denominator of T_e, i.e. $\cos(2\rho) = -1$ and $cosh(2\beta) \approx 1$. A second approach is to maximize its numerator by achieving $\beta \gg 1$ such that T_e is dominated by the hyperbolic terms of Eq. S13.

For the former condition, setting $\rho = \pi/2$, we get

$$h_e = \lambda/(4\mathrm{Re}\{\sqrt{\epsilon_e}\}). \tag{S14}$$

Using this value of h_e, we get $\beta = \pi\mathrm{Im}(n_e)/(2\mathrm{Re}(n_e))$. To assure $cosh(2\beta) \approx 1$, therefore $\beta \ll 1$, we see that the condition $\mathrm{Re}\{n_e\} \gg \mathrm{Im}\{n_e\}$ must be satisfied. This can be achieved outside the RB for frequencies $\omega < \omega_{TO}$. Furthermore, this leads to the spacer thickness approaching, in first order approximation:

$$h_m \approx \frac{\lambda}{2\aleph_m} \tag{S15}$$

For the second strategy, we set $\beta \gg 1$. So from Eq. S13, $T_e \approx i\mathrm{tanh}(2\beta)$ which leads to

$$n_e T_e \approx -\mathrm{Im}(n_e)\tanh 2\beta + i\mathrm{Re}(n_e)\tanh 2\beta$$
$$\frac{T_e}{n_e} \approx \frac{\mathrm{Im}(n_e)\tanh 2\beta + i\mathrm{Re}(n_e)\tanh 2\beta}{\mathrm{Im}(n_e)^2 + \mathrm{Re}(n_e)^2} \tag{S16}$$

We note that $\mathrm{Re}(n_e T_e)$ can be large only if $\mathrm{Im}(n_e) \gg 1$. As a result, $\mathrm{Im}(T_e/n_e) \ll 1$ and setting $r_n = 0$ in Eq. S10 leads to

$$\frac{\chi - \mathrm{Re}(n_e)\tanh 2\beta}{\mathrm{Im}(n_e)\tanh 2\beta} = \chi \frac{\mathrm{Im}(n_e)\tanh 2\beta}{\mathrm{Im}(n_e)^2 + \mathrm{Re}(n_e)^2} \tag{S17}$$

For this to be solvable, the LHS of Eq. S17 must be positive, implying $\mathrm{Re}\{n_\mathrm{e}\} \ll \mathrm{Im}\{n_\mathrm{e}\}$. This is true in the RB for frequencies $\omega > \omega_{TO}$. Solving Eq. S17 we get

$$h_\mathrm{e} = \frac{\lambda}{8\pi \mathrm{Im}\{\sqrt{\epsilon_\mathrm{e}}\}} \mathrm{arcsinh} \frac{2}{\cos\theta \mathrm{Re}\{\sqrt{\epsilon_\mathrm{e}}\}} \tag{S18}$$

Using this value of h_e, setting $r_n = 0$ in Eq. S10 we get in first approximation the same spacer thickness as derived previously in Eq. S15. Using the phase matching condition of Eq. S8, with $h_m = \frac{\lambda}{2\aleph_m}$ we get $\Psi = \pi$, which is the condition for directional emission (maximum FOM) as discussed in the main article. Also, we note that our strategy of maximizing $n_\mathrm{e} T_\mathrm{e}$ with $n_\mathrm{e} \gg 1$ results in $n_\mathrm{e} T_\mathrm{e} \gg T_\mathrm{e}/n_\mathrm{e}$. So the last terms of Eq. S10 and Eq. S12 can be neglected and we arrive at Eq. 5 and Eq. 6 of the main text.

To summarize, directional emission can be achieved either for $\mathrm{Re}(n_e) \gg \mathrm{Im}(n_e)$ or $\mathrm{Im}(n_e) \gg \mathrm{Re}(n_e)$, hence on both sides of the TO resonance. It should be noted that in first approximation, satisfying Eqs. S14, S18 for the emitter height and Eq. S15 for the spacer height ensures the FOM to be maximum. However, this derivation is only true for extreme cases when either $\mathrm{Re}\{n_\mathrm{e}\} \gg \mathrm{Im}\{n_\mathrm{e}\}$ or $\mathrm{Re}\{n_\mathrm{e}\} \ll \mathrm{Im}\{n_\mathrm{e}\}$. To maximize the FOM for intermediate cases, we can either fix $h_m = \lambda/2\aleph_m$ and deduce h_e from Eq. S8 or fix h_e and calculate the corresponding h_m. For the former, strong directionality is always achieved although peak emissivity will quickly decrease away from the optimal conditions. In the main article section 4, we fix h_e to ensure unitary emissivity and then we optimized h_m, observing a decrease in directionality (diffuse emission) away from the optimum. This results in the peaks of FOM as shown in Fig. 5c when $\Psi = \pi$ is satisfied.

We should also mention that the design rules presented in this article apply for all θ, however as the FOM scales with 2θ, the directionality is reduced when the structure is designed for lower angles of emission. The polar plots of the emissivity of 3 Salisbury screen structures optimized for 3 different angles at $\omega = 0.99\omega_{TO}$ are shown in Fig. S3.

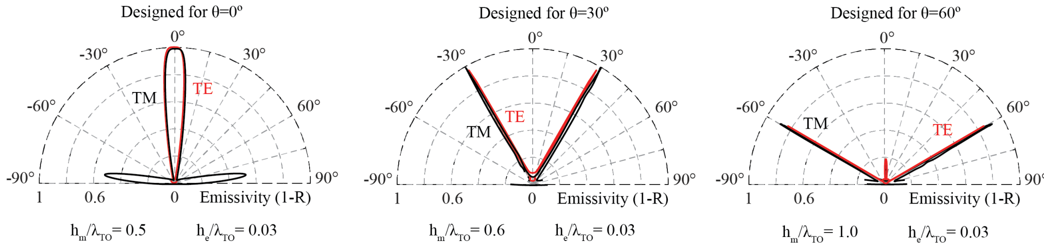

Fig. S3: Polar plot of the emissivity of optimized planar structures for both TM and TE polarized light, for three different angles of emission (θ). The optimized h_m and h_e for each structure are shown in the figure. The structures are optimized at $\omega = 0.99\omega_{TO}$.

4 Optimization of grating period

The in-plane wave-vector of SPhP is $k_{SP} = k_0\sqrt{[\epsilon_e - \epsilon_e^2]/[\alpha_{Ze} - \epsilon_e^2]}$. The SPhP mode is excited by a 2D grating when the grating condition is satisfied [1] for a grating period P given as

$$k_0 \sin\theta + k_{SP} - 2\pi/P = 0. \tag{S19}$$

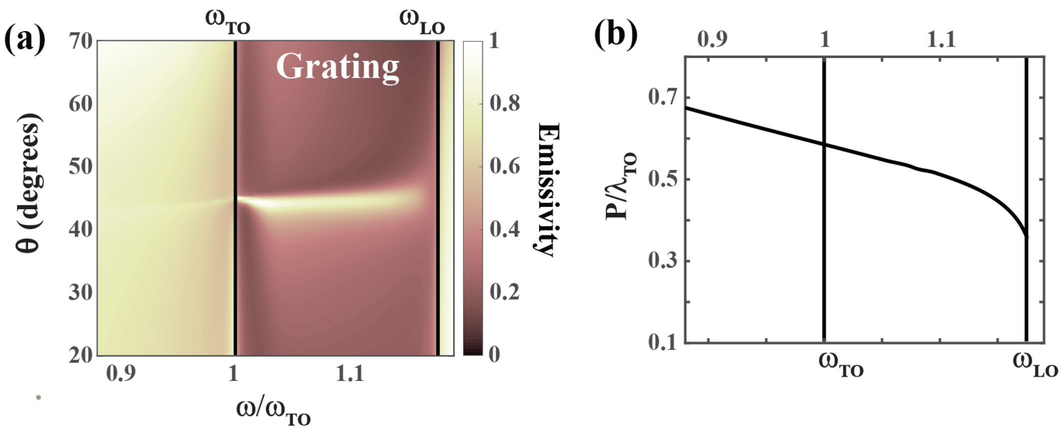

Fig. S4: (a) The calculated emissivity ($E = 1 - R$) as a function of ω (normalized to ω_{TO}) and θ for grating structure for TM polarized light. The grating period shown in (b) is optimized at each frequency to have directional emission at $\theta = 45°$.

The emissivity for a 2D grating on a semi-infinite substrate with permittivity ϵ_e was calculated numerically and is shown in Fig. 2d of the main article. The numerical calculations for the reflectivity of the grating structure are done by the Rigorous coupled wave analysis (RCWA) package by Hugonin and Lalanne [2]. We see almost unity emissivity for frequencies and incident angles corresponding to the grating equation (Eq.S19), only for TM polarized light. As SPhP are not supported for TE polarized light, the emissivity is negligible in the RB for TE polarization.

The grating height was fixed at $300nm$, which is close to the penetration depth of the SPhP in air. The grating period (P) was tuned according to Eq.S19 to have the SPhP resonance at $\theta = 45°$ for each frequency. This is shown in Fig. S4(b), normalized to the wavelength of the TO phonon resonance (λ_{TO}). The grating as expected has a narrow emission band around $\theta = 45°$ (Fig. S4(a)), only for the frequencies in the RB where the SPhP can be excited. The FOM for the grating shown in Fig.5c was calculated from the color-map shown in Fig. S4.

References

[1] Mitradeep Sarkar, Mondher Besbes, Julien Moreau, Jean-François Bryche, Aurore Olivéro, Grégory Barbillon, Anne-Lise Coutrot, Bernard Bartenlian, and Michael Canva. Hybrid Plasmonic Mode by Resonant Coupling of Localized Plasmons to Propagating Plasmons in a Kretschmann Configuration. *ACS Photonics*, 2(2):237–245, feb 2015.

[2] Jean Paul Hugonin and Philippe Lalanne. Reticolo software for grating analysis. *arXiv preprint arXiv:2101.00901*, 2021.
